# Risk factors associated with adverse perinatal outcome in planned vaginal breech labors at term: a retrospective population-based case-control study

**DOI:** 10.1186/s12884-017-1278-8

**Published:** 2017-03-20

**Authors:** Georg Macharey, Mika Gissler, Veli-Matti Ulander, Leena Rahkonen, Mervi Väisänen-Tommiska, Mika Nuutila, Seppo Heinonen

**Affiliations:** 1 Obstetrics and Gynaecology, University of Helsinki and Helsinki University Hospital, Helsinki, Finland; 2 THL National Institute for Health and Welfare, Helsinki, Finland

**Keywords:** Breech presentation, Risk factors, Cesarean section, Oligohydramnios, Fetal growth restriction, Gestational diabetes, Perinatal morbidity, Perinatal mortality, Macrosomia

## Abstract

**Background:**

Vaginal breech delivery is associated with adverse perinatal outcome. The aim of this study was to identify factors associated with adverse perinatal outcome in term breech pregnancies, and to provide clinicians an aid in selecting women for a trial of vaginal labor with the fetus in breech position.

**Methods:**

We conducted a retrospective, nationwide, Finnish population-based case-control study. All planned singleton vaginal deliveries at term with the fetus in breech position between the years 2005 and 2014 were analyzed. The study’s end point was a composite set of adverse perinatal outcomes. All infants with an adverse outcome were compared to the infants with normal outcomes. A multivariate logistic regression model was used to analyze the data.

**Results:**

An adverse perinatal outcome was recorded for 73 (1.5%) infants. According to the study results fetal growth restriction (adjusted odds ratio, 2.94; 95% CI, 1.30–6.67), oligohydramnios (adjusted odds ratio, 2.94; 95% CI, 1.15–7.18), a history of cesarean section (adjusted odds ratio, 2.94; 95% CI, 1.28–6.77, gestational diabetes (adjusted odds ratio, 2.89; 95% CI, 1.54–5.40), epidural anesthesia (adjusted odds ratio, 2.20; 95% CI, 1.29–3.75) and nulliparity (adjusted odds ratio, 1.84; 95% CI, 1.10–3.08) were associated with adverse perinatal outcome.

**Conclusions:**

Adverse perinatal outcome in planned vaginal breech labor at term is associated with fetal growth restriction, oligohydramnios, previous cesarean delivery, gestational diabetes, nulliparity and epidural anesthesia.

## Background

Breech presentation at term occurs in two to three per cent of all singleton term deliveries [1, 2]. The majority of breech pregnancies are delivered nowadays by cesarean section [3], as planned vaginal breech delivery is controversial. Some studies and most importantly the term breech trial by Hannah M. have reported that planned vaginal breech labor is associated with adverse perinatal outcome [4, 5]. Other studies have been published which show that vaginal breech delivery is safe for mother and child if the women for a trial of labor are carefully selected and labor management takes place in an appropriate obstetric setting [6, 7]. Many national associations of obstetricians and gynecologists have defined guidelines to determine under which circumstances a vaginal breech delivery is feasible [8–10], but even under these circumstances adverse perinatal outcomes still occur [11]. In recent literature there are only two articles that review risk factors associated with adverse perinatal outcome in planned vaginal breech delivery, but neither article had the statistical power to analyze risk factors for perinatal mortality and severe neonatal outcome [11, 12]. More evidence and a better understanding of factors associated with adverse perinatal outcome might aid in making optimal selections for a trial of vaginal breech labor. The aim of this study is to determine risk factors for adverse perinatal outcome in planned vaginal breech delivery, to aid practitioners in the selection of women for vaginal breech delivery and to limit the risks of adverse perinatal outcome.

## Methods

**Fig. 1:**
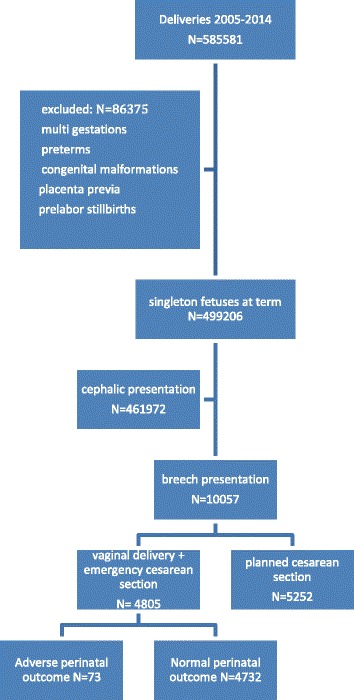
The data flow in the current study

The study was population-based, and the studied population included all deliveries from January 1, 2005 to December 31, 2014. It included all deliveries that had a trial of vaginal labor at term with a singleton fetus in breech presentation (frank, complete and incomplete breech presentation). The following pregnancies and infants were excluded from the study: Multiple gestations, preterm pregnancies, antepartum diagnosed stillbirths, placenta previa and infants with congenital malformations. Primary outcome was a composite index of perinatal and neonatal morbidity and mortality. Comparisons were made between all infants with adverse outcome and all infants with normal outcome. The flow chart of the selection process is shown in Fig. 1. Adverse perinatal outcome was defined as umbilical arterial pH < 7.00, five minute Apgar score below four and/or neonatal mortality during the first six days of life.

The study utilized the data of the national medical birth register and the hospital discharge register, maintained by the National Institute for Health and Welfare. Data for the medical birth register is collected at all maternity hospitals in Finland. Reporting to the national registers is obligatory, thus the data is valid and gives good, nationwide coverage [13]. The medical birth register includes all live births and stillbirths with a birth weight of 500 g or more or with a gestational age of 22 weeks or more. The hospital discharge register contains information on all inpatient periods in all Finnish hospitals and all outpatient visits recorded in the public sector. The registered information includes demographic data, maternal information before and after the delivery, intrapartum procedures and complications, as well as neonatal outcome. The information is coded according to the International Statistical Classification of Diseases and Related Health Problems 10th Revision (ICD-10).

Maternal factors, pregnancy factors and neonatal/fetal characteristics were studied. Maternal factors included parity, maternal age, smoking, bodymass index (BMI) before pregnancy, previous cesarean section as well as diabetes mellitus type one and two. Factors regarding the pregnancy were gestational age at delivery, gestational diabetes [defined as an abnormal two-hour oral glucose tolerance test, the test is diagnostic if one glucose level is elevated (Fasting glucose ≥ 5.3 mmol/L, or one hour ≥ 10.0 mmol/L, or two hour ≥ 8.5 mmol/L)], preeclampsia [defined as new onset of hypertension (≥140/90 mmHg) and proteinuria or hypertension and end-organ dysfunction with or without proteinuria after 20 weeks of gestation in a previously normotensive woman], assisted reproduction technology, oligohydramnios (defined as amniotic fluid index below 5 cm), epidural anesthesia and post term pregnancies. Neonatal and fetal characteristics included neonatal sex (male), birth weight, macrosomia defined as birth weight above 4500 g, fetal growth restriction defined as birth weight < 2SD. Statistical differences in categorical variables were evaluated with the chi-square test and differences in continuous variables by the Mann-Whitney U test as appropriate. Differences were deemed to be significant if *p* <0.05. In addition, 95% confidence intervals (CI) were calculated. The estimated risks (odd ratios (ORs) with their 95% CIs] of adverse outcomes were calculated using binary logistic regression. A multiple regression model was used for the adjusting. The data was analyzed using SPSS for Windows V.19.0, Chicago, Illinois, USA. The reporting of this study conforms to the STROBE statement. Authorization to use the data was obtained from the National Institute for Health and Welfare as required by national data protection legislation in Finland (Reference number THL/1200/5.05.00/2012).

## Results

During the study period of ten years 10 057 women went into labor with the fetus in breech presentation. This results in a breech presentation rate of 2% in singleton pregnancies at term, excluding pregnancies with congenital malformations. Out of these women 4805 women had a trial of vaginal breech labor, of which 3123 (65%) had a vaginal delivery and 1682 (35%) women had their delivery converted to a cesarean section.

Seventythree (1.5%) neonates had a severe adverse perinatal outcome (Table 1). The multivariable analysis showed that mothers of infants with adverse perinatal outcome were more often nulliparous (adjusted odds ratio [aOR], 1.84; 95% CI, 1.10–3.08), they had more often gestational diabetes (aOR, 2.89; 95% CI, 1.54–5.40), a history of cesarean section (aOR, 2.94; 95% CI, 1.28–6.77), they were more often diagnosed with oligohydramnios (aOR, 2.94; 95% CI, 1.15–7.18) and had more often epidural anesthesia (aOR, 2.20; 95% CI, 1.29–3.75). Among the neonatal factors fetal growth restriction (aOR, 2.94; 95% CI, 1.30–6.67) was significantly associated with an increased risk of adverse perinatal outcome. We identified four cases of uterus rupture in the study population, all of which had a history of cesarean section. Out of these four cases one neonate had an adverse perinatal outcome; three did not suffer from adverse outcome (odds ratio [OR], 21.89; 95% CI, 2.25–213.01) (Table 2).

**Table 1:** Adverse perinatal outcome in planned vaginal breech delivery

Perinatal outcome	Planned vaginal delivery *N* = 4805 *N* (%)
Early neonatal mortality < 7 d^a^	5 (0.1)
Umbilical arterial pH < 7.00^a^	42 (0.9)
Five min Apgar score < 4^a^	30 (0.6)
Combined adverse perinatal outcome^b^	73 (1.5)

^a^Criteria included in combined adverse perinatal outcome

^b^Infants with one or more factors

**Table 2:** Factors associated with adverse perinatal outcome in planned vaginal breech delivery

All vaginal deliveries and emergency breech cesarean sections at term (2005–2014)	Adverse outcome^a^ *N* = 73		Normal outcome *N* = 4732				
	N/mean	%/SD	N/mean	%/SD	*P* value	OR 95% CI	aOR 95% CI
Nulliparous							
Maternal age in years mean + SD	30.3	5.0	30.1	5.1	0.740		
BMI ≥ 30	9	10.7	233	7.7	0.303	1.45 (0.71–2.92)	
Smoking	16	19.0	421	13.9	0.176	1.46 (0.84–2.55)	
Diabetes mellitus type I	1	1.2	6	0.2	0.058	6.09 (0.73–51.16)	
Diabetes mellitus type II	0		6	0.2			
Preeclampsia	2	2.4	74	2.4	0.975	0.98 (0.24–4.05)	
Gestational age at delivery mean + SD	39.7	1.1	39.6	1.2	0.433		
Gestational diabetes	14	6.7	197	6.5	<0.001	2.89 (1.60–5.21)	2.89 (1.54–5.40)^b^
Fetal growth restriction^c^	7	8.3	87	2.9	0.004	3.09 (1.38–6.88)	2.94 (1.30–6.67)^b^
Assisted reproduction technology	3	3.6	50	1.6	0.178	2.21 (0.68–7.25)	
History of cesarean section	8	9.5	139	4.6	0.035	2.20 (1.04–4.64)	2.94 (1.28–6.77)^b^
Oligohydramnios	5	6.0	57	1.9	0.008	3.31 (1.29–8.49)	2.94 (1.15–7.81)^b^
Epidural anesthesia	65	77.4	1739	57.2	<0.001	2.56 (1.53–4.29)	2.20 (1.29–3.75)^b^
Post term pregnancies > 40	10	11.9	418	13.8	0.627	0.85 (0.43–165)	
Birth weight in grams	3293	431	3341	416	0.311		
Neonatal sex (male)	37	44.0	1367	45.0	0.865	0.96 (0.62–14.49)	
Uterus rupture	1	1.4	3	0.1	<0.001	21.89 (2.25–213.01)	NA^d^
Macrosomia (birth weight >4500 g)	4	5.5	428	9.0	0.291	0.58 (0.21–1.61)	

^a^Early neonatal mortality <7d, umbilical arterial pH < 7.00, 5 min Apgar score < 4

^b^Adjusted for nulliparous; gestational diabetes; fetal growth restriction (<−2SD); history of cesarean section; oligohydramnios; epidural anesthesia

^c^Fetal growth restriction defined as birth weight < 2SD

^d^ *NA* Not available

Maternal age, a BMI over 30, maternal smoking, average gestational age at delivery, diabetes mellitus type I an II, preeclampsia, neonatal weight, neonatal sex, birth weight, assisted reproduction technology, macrosomia and post term pregnancies were not associated with adverse perinatal outcome (Table 2).

## Discussion

Planned vaginal breech delivery at term is associated with adverse perinatal short-term outcome. The main findings of this study showed that nulliparity, gestational diabetes, fetal growth restriction, a history of cesarean section, oligohydramnios and epidural anesthesia were associated with adverse perinatal outcome in vaginal breech deliveries at term. Secondly, the frequency of severe perinatal outcome was with 1.6% (73 out of 4805 cases) much lower than in the term breech trail with 5.1% [4]. These results can be explained with a violation of the stringent criteria for vaginal breech delivery in the term breech trial, as in one third of all cases analyzed in the term breech trial the fetal sizes were considered small (<3000 g at term). Of all stillbirths and perinatal deaths notified in the term breech trial 69% (11 of 16 cases) were too small for their gestational age [4]. We identified fetal growth restriction (< −2SD /IUGR) also as a main risk factor for severe adverse perinatal outcome in this study. This result is coherent with a secondary analysis of the term breech trial data by Su, which showed that birth weight <2800 g was associated with adverse perinatal outcome [14]. Fetal growth restriction is a known risk factor for adverse fetal outcomes including stillbirth, cerebral palsy, neonatal death, and hypoxia ischemic encephalopathy [15–18]. Due to these risks many guidelines for vaginal breech delivery state fetal growth restrictions as a contraindication for a trial of vaginal labor [8–10].

We showed that adverse perinatal outcome was also associated with oligohydramnios. Oligohydramnios is associated with adverse pregnancy outcomes [19]. These adverse perinatal outcomes are most likely caused by umbilical cord compression, uteroplacental insufficiency and meconium aspiration [19]. Oligohydramnios is associated with reduced fetal movements [20], which is linked to adverse perinatal outcome [21–23].

The analysis showed that nulliparity was associated with adverse outcome in vaginal breech delivery. Other authors have already before shown that infants born to primigravid women have a higher rate of perinatal morbidity [24]. We found that the rate of women with gestational diabetes was also significantly higher in pregnancies with adverse perinatal outcome in vaginal breech delivery. Gestational diabetes has been associated with adverse perinatal outcomes before, in pregnancies with the fetus in cephalic presentation, as it increases perinatal mortality, the risk for macrosomia and preeclampsia [25]. A history of cesarean section was identified as a risk factor for adverse perinatal outcome. If the first cesarean section was due to dystocia, women have a higher risk to undergo another pathologic delivery [26]. It is also known that a trial of vaginal labor after cesarean section is associated with a higher perinatal mortality, birth asphyxia and sepsis [26]. The adverse outcome is mainly caused by uterine rupture and a prolonged labor [26, 27]. Our data confirms that a history of cesarean section is related to a higher risk of uterus rupture and that uterus rupture is associated with adverse perinatal outcome.

This study showed that women who received epidural anesthesia had a 2.2 times higher risk of adverse perinatal outcome. Earlier studies have shown that epidural anesthesia in connection with breech delivery is associated with a longer duration of labor and an increased need for augmentation of labor with oxytocin infusion [28]. It is not possible to tell whether the association between epidural anesthesia and adverse outcome is due to the epidural prolonging labor and delaying expulsion of the fetus or whether epidural anesthesia is simply used more in prolonged labors that inherently have a higher risk of expulsive delay and adverse outcome [11]. Interestingly macrosomia was not identified as a risk factor for adverse perinatal outcome in vaginal breech labors at term. However, macrosomia is associated with adverse perinatal outcome in cephalic presentation [29]. Dystocia related to macrosomia is a major concern in vaginal breech delivery at term and this concern is reflected in many guidelines. The guidelines of the Royal College of Obstetricians and Gynaecologists and the German guidelines suggest that fetuses in breech presentation with an estimated weight ≥ 3800 g should not be delivered vaginally [8, 10]. The Canadian guidelines suggest that fetuses ≥ 4000 g should not be delivered vaginally [9].

The main strength of this study is that it has the statistical power to generalize data regarding the outcomes of the infants. Another advantage of this study is that it is up to the author’s knowledge the largest population-based, case-control study reviewing risk factors in vaginal breech delivery at term. The study is based on nationwide data in a country, in which the medical treatment of pregnancies is very homogenous, as there are no private hospitals dealing with pregnancies. The study is limited by its retrospective design and due to a lack of information regarding whether the studied women had a history of breech presentation in previous pregnancies and a lack of information regarding the reason for previous cesarean sections. Additionally the studied variables were restricted to databank availability. The Finnish birth register lacked for example records of neonatal cord arterial base deficit. Infants in breech position born vaginally often suffer from significant respiratory acidosis due to cord compression from which they easily recover by ventilation. It would be important to differentiate these cases from cases with significant metabolic acidosis, as metabolic acidosis is probably the best early indicator for a higher risk of adverse neurodevelopmental outcome. Unfortunately, this data was not available and therefore such an analysis was beyond the scope of the present study and warrants attention in future studies.

## Conclusions

The study confirmed fetal growth restriction as a risk factor for adverse perinatal outcome. Oligohydramnios, nulliparity, gestational diabetes, epidural anesthesia and a history of cesarean section were identified as new risk factors for adverse perinatal outcome in a trial of labor in pregnancies with the fetus in breech position. These factors should be taken into account when counseling women and included in all national guidelines for vaginal breech delivery. In particular, vaginal delivery with the fetus in breech presentation should be avoided when fetal growth is restricted. Macrosomia as an individual risk factor for adverse perinatal outcome should be reviewed; further studies are needed to investigate this issue. A more careful selection of the women eligible for vaginal breech delivery has the potential to reduce the risk of adverse perinatal outcome. Caution and a willingness to abandon a trial of labor if circumstances are not favorable are essential for the safety of mother and child.

## Abbreviations

aOR: Adjusted odds ratio
BMI: Body mass index
CI: Confidence interval
ICD: International classification of diseases
OR: Odds ratio
THL: National Institute for Health and Welfare
